# Response of the Arabidopsis indolic secondary metabolite network to infection with *Colletotrichum higginsianum*

**DOI:** 10.3389/ffunb.2026.1761006

**Published:** 2026-03-16

**Authors:** Julia Seufer, Dorota Speer, Rebekka aus den Erlen, Erich Glawischnig, Lars M. Voll

**Affiliations:** 1 Molecular Plant Physiology, Department Biology, Marburg University, Marburg, Germany; 2 Microbial Biotechnology, Technical University of Munich, Straubing, Germany; 3 Center for Synthetic Microbiology (SYNMIKRO), Marburg University, Marburg, Germany

**Keywords:** Arabidopsis, camalexin, *Colletotrichum higginsianum*, indole carboxylic acid, indole glucosinolates, indole metabolism, host defence

## Abstract

In plants, the indolic amino acid tryptophan (Trp) serves as the precursor for the synthesis of a plethora of indolic secondary metabolites with allelopathic activity against microbes or toxicity against herbivores. In the cruciferous model plant Aradidopsis, indolic glucosinolates, the major phytoalexin camalexin, carbonyl nitriles and indole carboxylic acids are abundant products of the branched biosynthetic pathway that originates from the Trp oxidation product indole-acetaldoxime (IAOx). To date, it has not been intensely investigated, (i) how the Arabidopsis indolic metabolic network responds to fungal infection and (ii) which indolic metabolites play a role for compatibility upon fungal attack. To provide a systematic case study, we have employed a combination of single, double, triple and quadruple Arabidopsis mutants lacking selected combinations of indolic metabolites for leaf infections with the hemibiotrophic ascomycete *Colletotrichum higginsianum*. Our study revealed that only camalexin, but neither indolic glucosinolates nor the recently described phytoalexin 4-hydroxy-carbonyl nitrile (4-OH-ICN) had a significant role for the resistance towards *C. higginsianum*. Besides its known relevance during the late necrotrophic phase, our data suggest a role of camalexin in early post-penetration defense. Our study also indicates that, indole acetonitrile (IAN) can be produced upon cleavage of indolic glucosinolate by myrosinases in pathogen challenged leaves and feed into camalexin biosynthesis in case further IAN conversion by CYP71B6 is blocked. Downstream of IAN, we found that AAO1 and CYP71B6 act redundantly in the accumulation of indole carboxylic acid (ICOOH). We also revealed that *CYP71A12* has a stronger contribution to camalexin biosynthesis in *C. higginsianum* infected leaves than in previously investigated abiotic stress models. While our dataset suggests clear, but subtle differences in the response of the indole metabolic network in pathogen and abiotic challenge, we can rule out a contribution of the fungal pathogen to the observed differences due to our study design.

## Introduction

**Figure 1:**
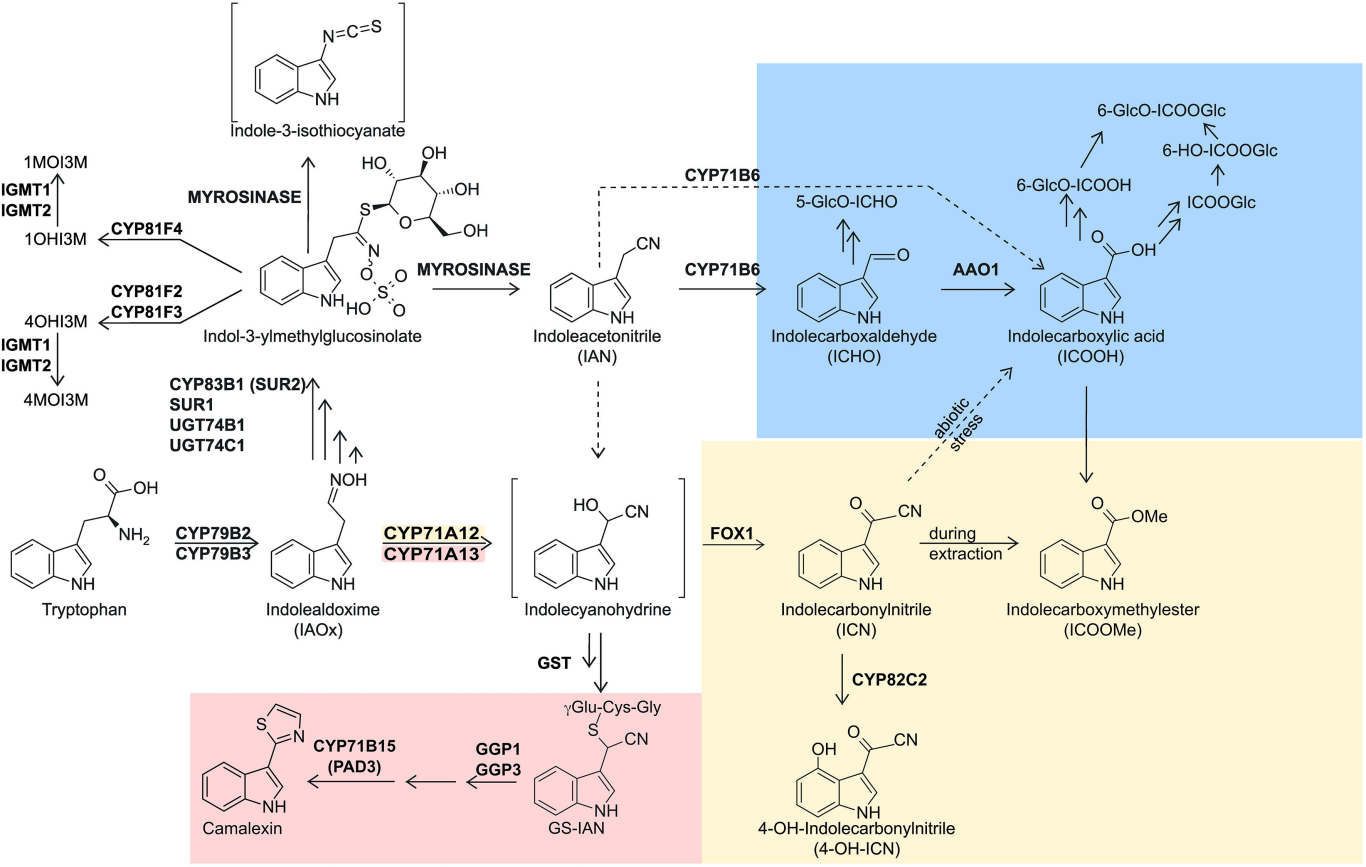
Overview over the indolic metabolic network downstream of tryptophan. The camalexin branch (red), the indole carbonylnitrile branch (yellow) and the indole carboxylate branch (blue) are indicated by coloured boxes, the glucosinolate branch is shown blank. Routes only present in biotic or abiotic stress (additionally labeled) are depicted by dashed arrows (see discussion section for details). Metabolite abbreviations are indicated in the figure, except for GS-IAN, glutathione-indole-3-acetonitrile. Gene abbreviations are as follows: AAO, aldehyde oxidase; CYP, cytochrome P450 monooxygenase; FOX1, flavin-dependent oxidoreductase; GGP, gamma-glutamyl peptidase; IGMT, indole glucosinolate methyl transferase; SUR, S-alkyl-thiohydroximate lyase SUPERROOT; UGT, UDP-glucosyl transferase.

Indolic metabolites play crucial roles in the interaction of cruciferous plants with pathogens and herbivores (Halkier and Gershenzon, 2006; Rauhut and Glawischnig, 2009) and represent a diversified class of secondary plant products comprising phytoanticipins and phytoalexins (Pedras et al., 2011). The aromatic amino acid tryptophan is the entry point for the biosynthesis of indolic defense compounds in the crucifer model plant *Arabidopsis thaliana*. Conversion of tryptophan (Trp) to the precursor indole-3-acetaldoxime (IAOx) by the CYP450 monooxygenases CYP79B2/CYP79B3 represents the committed step at the entry point to the network of indolic secondary metabolites (Hull et al., 2000; Mikkelsen et al., 2000; Zhao et al., 2002; Glawischnig et al., 2004; Böttcher et al., 2009; **Figure 1**). In Arabidopsis, major outputs of the network are indolic glucosinolates (IGSL) that are formed from the branch initiated by the CYP450 monooxygenase CYP83B1 (Bak et al., 2001; Hansen et al., 2001; represented black in **Figure 1**), while the closely related IAOx dehydratases CYP71A12 and CYP71A13 initiate the branch towards indolic phytoalexins (e.g. Müller et al., 2015), with CYP71A13 being predominantly involved in the biosynthesis of the major Arabidopsis phytoalexin camalexin (e.g. Nafisi et al., 2007; represented in red in **Figure 1**), while CYP71A12 is more important for channeling IAOx into the more recently discovered phytoalexin 4-hydroxyindole-3-carbonyl nitrile (4-OH-ICN; represented in beige in **Figure 1**) and derivatives of indole carboxylic acid (ICOOH) (Rajniak et al., 2015; Müller et al., 2015; represented in blue in **Figure 1**). Processivity from Trp towards camalexin is enhanced by the formation of a metabolon at the ER membrane, comprising all involved CYP450 monooxygenases as well as additional enzymes of the pathway (Mucha et al., 2019). Despite the existence of this metabolon, the product of the IAOx dehydratases CYP71A12 and CYP71A13, Indole-3-acetonitrile (IAN), was observed to be released by the pathway (Nafisi et al., 2007; Müller et al., 2015), but IAN can also arise from the degradation of indolic glucosinolates (IGSL) (de Vos et al., 2008). IAN can be converted to indole-3-carbaldehyde (ICHO) by the CYP450 monooxygenase CYP71B6 (Böttcher et al., 2014), and represents an alternative entry point to the biosynthesis of ICOOH derivatives after subsequent oxidation by ALDEHYDE OXIDASE1 (AAO1) (Böttcher et al., 2014; Müller et al., 2019).

Besides the long-known function of IGSL as phytoanticipins and their involvement in the biochemistry of the mustard oil bomb (Lüthy and Matile, 1984), there is ample genetic and biochemical evidence that degradation of IGSL by the atypical myrosinase PEN2 represents the committed step towards the formation of phytoalexins that are active against a wide range of bacterial, oomycete and fungal pathogens (e.g. Bednarek et al., 2009; Clay et al., 2009; Nafisi et al., 2007; Schlaeppi et al., 2010; Frerigmann et al., 2016; Stahl et al., 2016). In response to fungal infections, accumulation of the major phytoalexin camalexin (Tsuji et al., 1992) is usually required for full resistance (e.g. Zhou et al., 1999; Thomma et al., 1999; Bohman et al., 2004; O’Connell et al., 2004; Schlaeppi et al., 2010; Fuchs et al., 2016; He et al., 2019; Ferrari et al., 2007) and there is indication that camalexin is more toxic to fungal than to bacterial pathogens (as compiled by Nguyen et al., 2022). In summary, camalexin represents a crucial component in the defense against hemibiotrophic and necrotrophic fungi (e.g. Zhou et al., 1999; Thomma et al., 1999; Bohman et al., 2004; Narusaka et al., 2004; Staal et al., 2006; Chanda et al., 2008). In contrast to IGSL and camalexin, it remains elusive if indole carboxylic acids serve as phytoalexins. No direct antifungal activity has been shown for ICOOH towards *Plectosphaerella cucumerina* hyphae *in vitro* (Gamir et al., 2018). In addition, *Botrytis cinerea* detoxifies camalexin via IAN to ICOOH (Kliebenstein et al., 2005; Pedras et al., 2011), while *Rhizoctonia solani* detoxifies camalexin to IAN (Pedras and Khan, 1997), suggesting that metabolism of indolics via CYP71B6 and AAO1 does not serve the biosynthesis of alternative phytoalexins.

In the past, induction of indolic metabolism by harsh abiotic stresses like heavy metal or UV treatment have been predominantly employed to elucidate the plasticity of the indolic metabolite network (Böttcher et al., 2014; Müller et al., 2015, 2019). In the background of the camalexin defective *cyp71a12/cyp71a13* double mutant, additional deficiency in *CYP71B6* and *AAO1* resulted in largely additive penalties on the accumulation of indole carboxylic acid derivatives upon silver nitrate and UV exposure (Müller et al., 2019). While the accumulation of free ICHO was additively increased in the same set of mutants upon UV treatment only, *CYP71A13* and *CYP71B6* were additive for the accumulation of 5-(β-glucosyloxy)-indole-3-carbaldehyde (5-OGlc-ICHO), but not 4-(β-glucosyloxy)-indole-3-carbaldehyde (4-OGlc-ICHO), upon both silver nitrate and UV (Müller et al., 2019). This heterogeneity in the response of individual compounds towards these two abiotic stress regimes indicates that multiple stimuli are integrated into the response of the indolic metabolite network.

To date, only one study provides some information on the response of the Arabidopsis indole network in response to phytopathogen infection (Pastorczyk et al., 2020). Similar to what was observed in silver nitrate and UV elicited plants (Böttcher et al., 2014; Müller et al., 2015, 2019), CYP71A12 was vital for the pathogen-triggered accumulation of ICOOH derivatives upon infection with the fungal necrotroph *P. cucumerina* (Pastorczyk et al., 2020). The same study found that IGSL hydrolysis to IAN represented a negligible source of ICOOH derivatives in *P. cucumerina* infected leaves, but the contribution by *CYP71B6* and *AAO1* was not tested as the study had a different focus (Pastorczyk et al., 2020). In this interaction, PEN2-depdendent formation of phytoalexins was important for pre-invasion resistance, while camalexin deficient *cyp71a12/cyp71a13* exhibited strongly reduced post-penetration resistance, both against the necrotrophic pathogen *P. cucumerina* and the non-adapted hemibiotroph *Colletotrichum tropicale* (Pastorczyk et al., 2020).

In the Arabidopsis–*Colletotrichum higginsianum* interaction studied here, fungal conidia land on the leaf surface, produce germ tubes, which then differentiate dome-shaped, melanized appressoria (O’Connell et al., 2004; Engelsdorf et al., 2013). During maturation, compatible solutes accumulate inside the appressoria, while rigidity of appressorial cell walls increases (Mengden and Hahn, 2002; Münch et al., 2008). External water supply by surrounding water droplets results in the establishment of a high turgor pressure inside the appressoria that is instrumental for piercing the cell wall of the underlying epidermis cell with a penetration peg by a combination of mechanical force and lytic enzyme activities (Bechinger et al., 1999; Deising et al., 2000). In the penetrated host epidermis cells, *C. higginsianum* establishes itself as a biotroph within 36 h post inoculation by initially forming a bulbous infection vesicle that subsequently produces lobed biotrophic primary hyphae (O’Connell et al., 2004; Engelsdorf et al., 2017). Upon the subsequent colonization of neighboring cells at around 72 h post inoculation, a switch in both hyphal morphology and lifestyle occurs. Narrow-bore necrotrophic secondary hyphae grow rapidly and hyphal spread will eventually lead to necrotic lesions on the infected leaves that are visible shortly after the switch to necrotrophy (Mendgen and Hahn, 2002).

ROS accumulation and callose papilla formation in the host were shown to be largely irrelevant for resistance towards *C. higginsianum* (Birker et al., 2009), eliminating bias by these two major basal defense responses from the resistance readout. Previous work already provided indication that camalexin is required for full resistance towards *C. higginsianum* during necrotrophic colonization (Narusaka et al., 2004; Chanda et al., 2008), but the *pad3* mutant used in these studies accumulates the penultimate precursor dihydrocamalexin (DHCA) instead of camalexin to high amounts (Böttcher et al., 2009), for which antimicrobial activity has also been reported (Kempthorne et al., 2021). Similarly, an imbalance between the IGSL and phytoalexin branch resulted in diminished resistance towards *C. higginsianum* during necrotrophic colonization, but the accumulation of indoles from the phytoalexin branch had not been quantified (Engelsdorf et al., 2017).

Based on the available data, we hypothesized that IGSL contribute to the resistance against *C. higginsianum*, while indole carboxylates were unlikely to do so. Therefore, we employed a comprehensive set of higher order mutants in the phytoalexin branch (as described by Müller et al., 2019) that lack camalexin, but produce different bouquets of other indolic substances. However, the response of the indolic metabolic network towards *C. higginsianum* infection was unknown and only very limited information on the response of the network in other fungal pathosystems was available. By analyzing the levels of key indolic substances in response to *C. higginsianum* infection in the investigated mutants, our study was also set out to close this knowledge gap and would eventually allow to compare the *C. higginsianum* induced dynamics of the metabolic network to the available data from abiotic stresses (Böttcher et al., 2014; Müller et al., 2015; 2019) and *P. cucumerina* infection (Pastorczyk et al., 2020).

## Materials and methods

### Plant and fungal material and growth conditions

Arabidopsis plants were grown for three weeks in a 12 h light (22 °C)/12 h dark (20 °C) cycle in GroBanks (CLF Plant Climatics, Germany) at 110 µE·m^−2^·s^−1^ PFD. Seven days prior to inoculation with *C. higginsianum*, each plant was fertilized with 40 ml 0.1% WUXAL-Super fertilizer (Aglukon, Düsseldorf, Germany).

All mutants are situated in the Col-0 background with the insertion alleles as indicated below. All other mutant lines except *cyp82c2* (GABI_261D12) were taken from Müller et al. (2019) and screened by PCR as described therein: *aao1* (SALK_069221), *cyp71a12* (GABI_127H03), *cyp71a13* (SALK_105136), *cyp71b6* (GABI_305A04), *aao1/cyp71b6*, *aao1/cyp71a12/cyp71a13*, *cyp71b6/cyp71a12/cyp71a13*, *aao1/cyp71b6/cyp71a12/cyp71a13*. Seeds for *cyp82c2* (GABI261D12) were obtained from the NASC stock center and screened for homozygosity. The *cyp71a12/cyp71a13/cyp82c2* triple mutant was obtained by crossing the *cyp71a12/cyp71a13* double mutant (Müller et al., 2015) with *cyp82c2* (GABI_261D12).

*Colletotrichum higginsianum* isolate MAFF 305635 (kindly provided by the Ministry of Forestry and Fisheries, Japan) was grown on oat meal agar plates (OMA: 5% (w/v) shredded oat meal, 1.2% (w/v) agar) for 7 days at 22 °C under 60 µE·m^−2^·s^−1^ PFD illumination to promote conidia formation.

### *C. higginsianum* infection assays

Leaf infection by *C. higginsianum* was performed by spray inoculation of 4-week-old plants with a conidia titer of 2·10^6^ conidia/ml as described by Voll et al. (2012). Fungal colonization during the necrotrophic stage at 4 dpi was determined after pooling leaf punches from the three youngest fully expanded leaves of the same plant per replicate, following the qPCR-based procedure as described by Engelsdorf et al. (2013), while fungal entry rate at 2 dpi was assessed by trypan blue staining of the youngest fully expanded leaf as described by Engelsdorf et al. (2017). Samples for quantifying indolic metabolites were always taken at the same time point during the early biotrophic phase (2 dpi) from the same set of plants. Based on our previous experience (Engelsdorf et al., 2013, 2017; Gebauer et al., 2017; McCollum et al., 2019; Schmidt et al., 2020), quantification of primary and secondary metabolites after the onset of necrotrophic colonization at 2.5 dpi is strongly biased by the degree of colonization and can result in misleading readouts.

### Extraction of indolic metabolites

Samples for quantification of indolic metabolites were taken at 2 dpi from water-sprayed mock and *C. higginsianum* inoculated four-weeks old plants. To this end, the three youngest fully expanded leaves were pooled and snap-frozen in liquid N_2_. The harvested plant material was weighed and homogenized in a precooled Retsch adapter at 20 Hz for 1 min in a Retsch MM200 mill after the addition of 300 µl −20 °C pre chilled 80:20 MeOH:H2O and 5 µl of 1 mM Biochanin A as an internal standard. The plant material was extracted at 65 °C for 10 min. To remove any particles, the extract was centrifuged twice for 10 min prior to LC-MS analysis.

### LC-MS analysis of indolic metabolites

LC-MS analysis of indolic metabolites was essentially conducted as described by Müller et al. (2019). Indolic metabolites were quantified by separating 5 µL of each methanolic extract on a Macherey-Nagel NUCLEODUR C18 ec column (100 Å, 3 µm, 125 × 2.0 mm) held at 50 °C at a flow rate 0.2 mL min^−1^. Mobile phase A was water with 0.1% formic acid; mobile phase B was acetonitrile with 0.1% formic acid. A linear gradient was applied as follows: 0–30 min, 3–50% B linear; 45–50 min 50–97% B linear; 97% B (isocratic); 51–55 min, 3% B (isocratic). UV absorbance was monitored at 220 nm. The LC was coupled to a Thermo Scientific Q Exactive Plus operated in positive electrospray ionization (ESI) mode. Eluting compounds were detected by mass scanning from m/z 100–750. Thermo RAW files were converted to centroided mzXML using ProteoWizard msconvert (version 4.1.136, vendor peak picking for MS1 and MS/MS) to allow batch processing. Subsequent data processing and analysis was performed in Python (3.12.3) using pyOpenMS with custom scripts. The m/z fragments that were used for quantitation are listed in **Supplementary Table 1**.

### Glucosinolate extraction and UHPLC analysis of desulfoglucosinolates

Samples for quantification of glucosinolates were taken at 2 dpi from water-sprayed mock and *C. higginsianum* inoculated four-weeks old plants.

Glucosinolates were extracted and quantified from pooled 0.5 cm^2^ leaf punches of the three youngest fully expanded leaves, as described in Engelsdorf et al. (2017) with minor modifications. Leaf material was extracted twice with 1 ml 80% (v/v) methanol with one addition of 20 µl 5 mM benzyl glucosinolate as an internal standard. The two methanol extractions were combined and applied to DEAE Sephadex A-25 columns equilibrated with 0.5 M acetic acid/NaOH pH 5, and washed five times with 0.5 ml water and two times with 0.5 ml 20 mM acetic acid/NaOH, pH 5. After addition of 50 µl purified *Helix pomatia* sulfatase (EC 3.1.6.1, type H-1, 16–400 U g^–1^,Sigma, Deisenhofen, Germany), columns were sealed and left for overnight digestion. The resulting desulfoglucosinolates were eluted by adding three times 400 µl HPLC water. The eluate was lyophilized and resuspended in 200 µl HPLC water. Samples were analyzed by UHPLC on an Agilent 1260 Infinity system (Agilent, Santa Clara, USA). For the HPLC analysis, 50 µl desulfoglucosinolates were applied to a Macherey-Nagel EC250/4 Nucleosil 100–5 C18 and eluted using the following elution program with solvents A (water) and B (ACN). 0–10 min 3% B isocratic. 10–15 min 3–20% B. 15–30 min isocratic 20% B. 33–50 min Wash 97% B. 50–52 min 97-3% B. 52–62 min 3% B equilibration.

Elution was operated at 0.6 ml·min^–1^ flow and a column temperature of 25 °C. Analytes were detected at 229 nm and quantified based on response factor and internal benzylglucosinolate standard, as described previously (Gigolashvili et al., 2007).

### Statistical analysis and software

Figures were generated in Python (3.12.3) using matplotlib/seaborn, with bar charts showing group means ± SEM and box plots showing the median and IQR with whiskers at 1.5×IQR. The number of biological replicates is indicated in the respective figure legends. For fungal colonization, biological replicates from all experimental replications were pooled for statistical analysis. Statistical analyses and compact letter displays (CLD) were performed in R (4.3.2). Depending on the analysis, we used Fisher’s LSD (agricolae::LSD.test), Tukey’s HSD (agricolae::HSD.test) with α = 0.05. CLD letters indicate groups without significant differences and were placed above the corresponding bars/boxes. Genotypes were treated as categorical factors and presented in a predefined order.

Chemical structure depictions were drawn with Marvin (ChemAxon; https://chemaxon.com) and finalized in Inkscape.

## Results

### Loss of *CYP71A13* results in pathogen-dependent accumulation of indolic glucosinolates

To obtain comprehensive insight how the metabolic network downstream of indole-3-acetaldoxime (IAOx) responds to fungal infection with *C. higginsianum*, we included the *cyp17a12/cyp71a13* mutant, which is entirely devoid in camalexin production (Müller et al., 2015), and triple and quadruple mutants additionally defective in *cyp71b6* and/or *aao1*, that are compromised in various steps of the conversion from indole-3-acetonitrile (IAN) to indole-3-carbaldehyde (ICHO) and further downstream to indole carboxylic acids (Müller et al., 2019). We also included a *cyp17a12/cyp71a13/cyp82c2* triple mutant that was supposed to lack the production of 4-hydroxy-carbonyl nitrile (4-OH-ICN) in addition to camalexin (Rajniak et al., 2015). All respective single and double mutants served as controls. Besides the wild type, *cyp79b2/cyp79b3* and *pad3* were included as benchmarks.

To assess as to whether the balance between the IG and the phytoalexin branch is altered in the examined mutant portfolio, we quantified the levels of IGs by HPLC as described by Engelsdorf et al. (2017), while indoles and camalexin were quantified by LC-MS as described in the methods section. Indole carboxylic acid (ICOOH) and camalexin quantification was backed up by HPLC analysis. Previous studies had shown that altered susceptibility commonly only resulted in minimal differences in fungal biomass between genotypes during the initial biotrophic phase, while differences in fungal colonization could differ up to 20-fold after the onset of rapid fungal expansion during the necrotrophic phase (e.g. Engelsdorf et al., 2013; McCollum et al., 2019). In order to minimize bias in metabolite content by the degree of fungal colonization, we therefore sampled infected plant material at 2 dpi, which corresponds to the initial biotrophic phase (Engelsdorf et al., 2013, 2017; McCollum et al., 2019; Schmidt et al., 2020).

**Figure 2:**
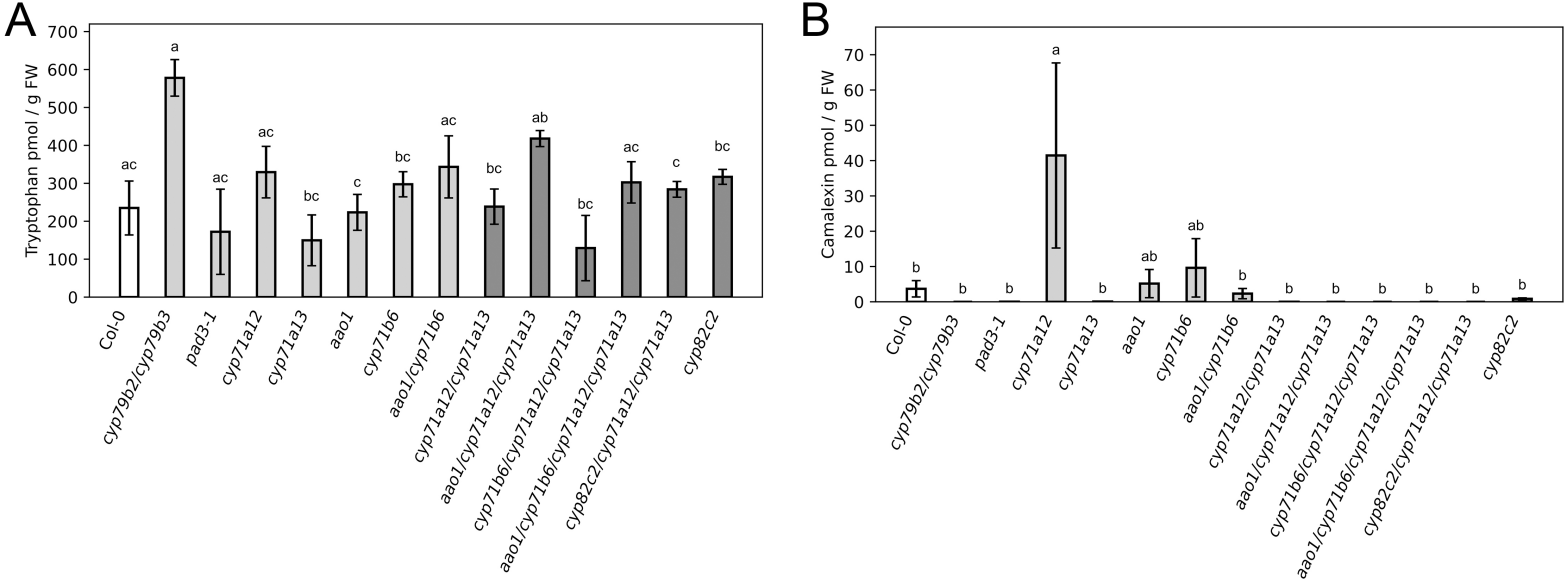
Foliar content of the precursor Tryptophan and the phytoalexin camalexin in mock conditions. Content of **(A)** tryptophan and **(B)** camalexin in mock treated leaves from four week-old plants are shown at two days post treatment. Wild type control (white) and *cyp71a12/cyp71a13* double and higher order mutants (dark gray) differ in color from the rest of the genotypes (light gray). Values are means of six biological replicates ± SEM. For each replicate, the three youngest fully expanded leaves from one single plant were pooled. Genotypes are indicated below the figure. Statistical analysis was conducted with a two-way ANOVA and a Fisher LSD *post hoc* test with letters indicating significant differences.

First, we confirmed that the phytoalexin camalexin was largely absent in mock treated leaves (**Figure 2**). In these conditions, the content of the precursor tryptophan ranged around 50 nmol/g FW in most genotypes, except for *cyp79b2/cyp79b3*, which showed a 2-fold increase in Trp compared to wild type (**Figure 2**). Similarly, the contents of the two detected IGSL, Indol-3-yl-methyl glucosinolate (I3M) and 4-Methoxyindole-3-ylmethyl glucosinolate (4MOI3M) were comparable in most genotypes in mock conditions (**Supplementary Figure 1**).

**Figure 3:**
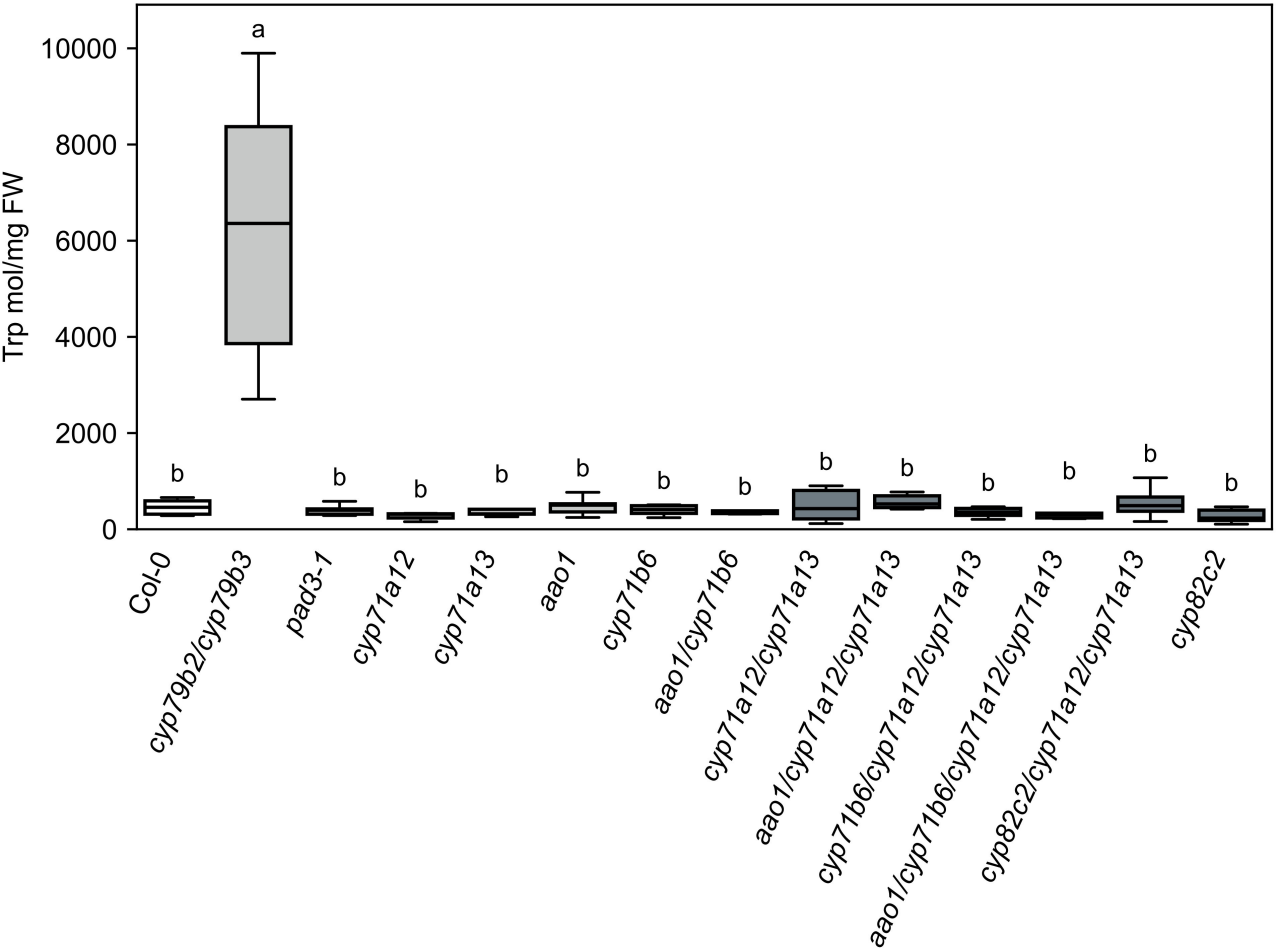
Tryptophan content at 2 days post infection (dpi) with *C. higginsianum*. Four week-old plants were spray inoculated with 2 · 10^6^ conidia/ml at the end of the light phase and foliar content of tryptophan are shown at two days post infection (dpi). Wild type control (white) and *cyp71a12/cyp71a13* double and higher order mutants (dark gray) differ in color from the rest of the genotypes (light gray). Box plots show the median and interquartile range (IQR) of six biological replicates with whiskers at 1.5×IQR. For each replicate, the three youngest fully expanded leaves from one single plant were pooled. Genotypes are indicated below the figure. Statistical analysis was conducted with a two-way ANOVA and a Fisher LSD *post hoc* test with letters indicating significant differences.

**Figure 4:**
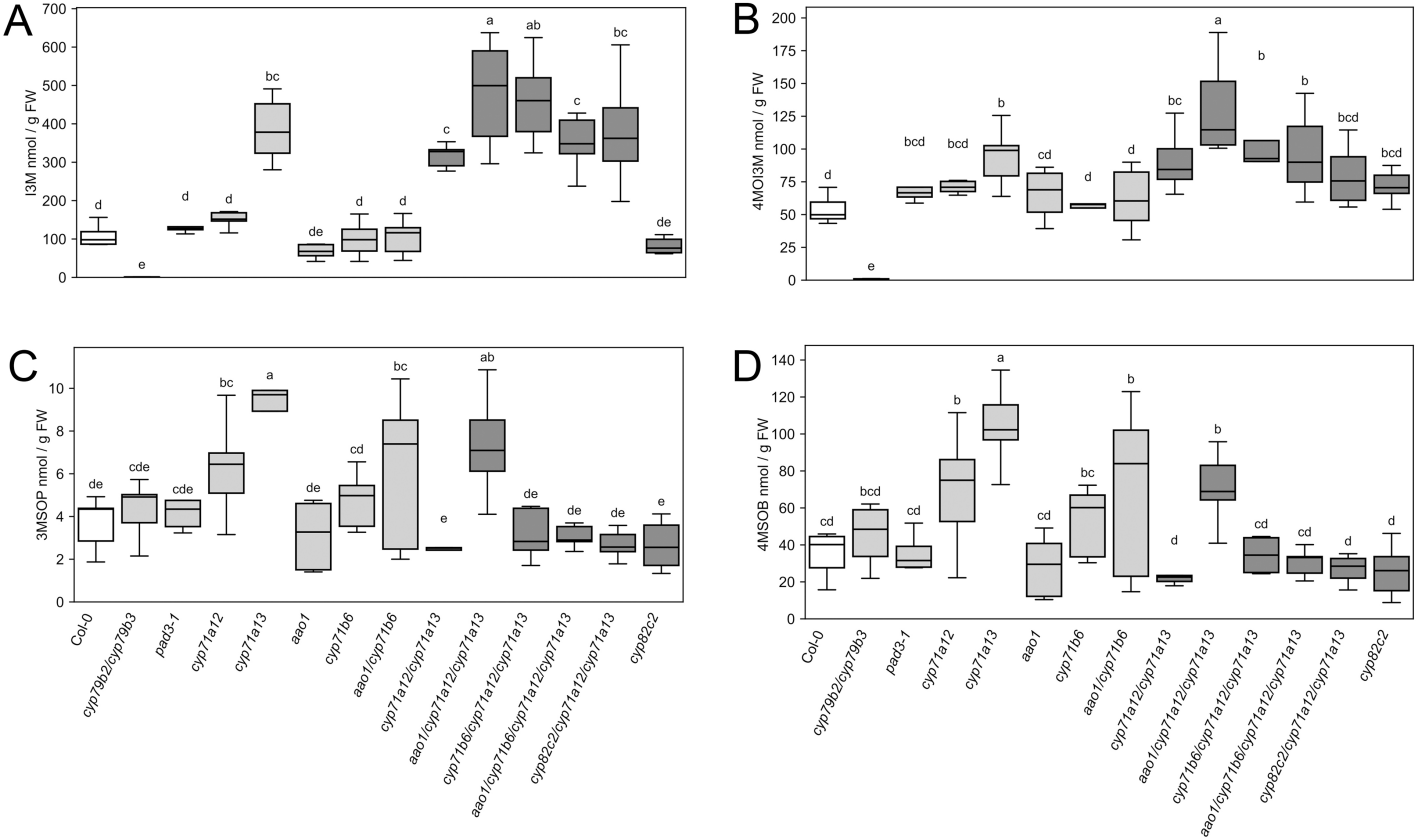
Foliar content of indolic and major aliphatic glucosinolates at 2 days post infection (dpi) with *C. higginsianum*. 4-week-old plants were spray inoculated with 2 · 10^6^ conidia/ml at the end of the light phase and foliar content of the indolic glucosinolates **(A)** I3M and **(B)** 4MOI3M, as well as the two major aliphatic GSL **(C)** 3MSOP and **(D)** 4MSOB are shown at two days post infection (dpi). Wild type control (white) and *cyp71a12/cyp71a13* double and higher order mutants (dark gray) differ in color from the rest of the genotypes (light gray). Box plots show the median and interquartile range (IQR) of six biological replicates with whiskers at 1.5×IQR. For each replicate, the three youngest fully expanded leaves from one single plant were pooled. Genotypes are indicated below the figure. Statistical analysis was conducted with a one-way ANOVA and a Fisher LSD *post hoc* test with letters indicating significantly different GSL contents. For data from mock treated plants, please see **Supplementary Figure 1**.

At 2 days post infection, tryptophan content increased to around 200 nmol/g FW in most genotypes, while the *cyp79b2/cyp79b3* double mutant that is unable to convert Trp to IAOx accumulated more than 6 µmol/g FW Trp (**Figure 3**). Unlike the previous observations for abiotic stress treatments (Müller et al., 2019), all genotypes lacking *cyp71a13* accumulated 3–4-fold more I3M than the wild type at 2 dpi (**Figure 4A**). Accumulation of 4MOI3M at 2 dpi ranged around 1.5–2-fold relative to wild type in *CYP71A13* deficient genotypes and was less pronounced than I3M accumulation (**Figure 4B**). Interestingly, I3M levels were diminished in genotypes lacking *cyp17a12/cyp71a13* in mock conditions (**Supplementary Figure 1**). Triple mutants lacking *cyp17a12/cyp71a13* as well as *cyp71b6* or *cyp82c2* showed an almost two-fold reduction in I3M in mock treated compared to infected leaves (**Supplementary Figure 1**).

Relative to wild type, the contents of the two major aliphatic GSL, 3MSOP and 4MSOB, were reduced by more than 50% in *aao1*, *cyp71b6*, *cyp82c2* and *cyp71a12/cyp71a13* in mock control conditions (**Supplementary Figure 1**), which was less pronounced at 2 dpi (**Figures 4C, D**). The response of indolic and aliphatic GSL towards *C. higginsianum* infection did not follow an obviously similar pattern in any of the mutants, indicating the absence of a strong crosstalk between indolic and aliphatic GSL production.

### Blocking IAN conversion by CYP71B6 reinstalls formation of camalexin in *cyp71a12/cyp71a13* double mutants at very low rates

**Figure 5:**
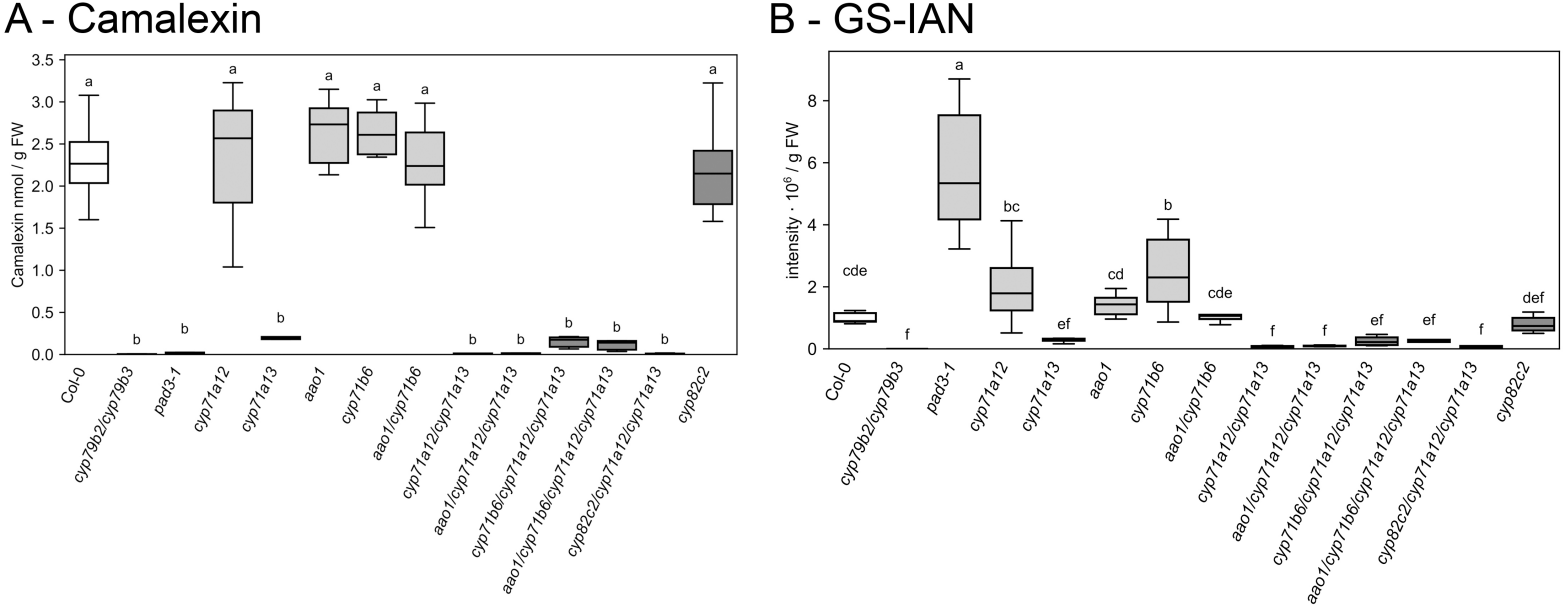
Foliar content of camalexin and GS-IAN (glutathione-indole-3-acetonitrile) at 2 days post infection (dpi) with *C. higginsianum*. 4-week-old plants were spray inoculated with 2 · 10^6^ conidia/ml at the end of the light phase and foliar content of **(A)** camalexin and **(B)** GS-IAN are shown at two days post infection (dpi). Wild type control (white) and *cyp71a12/cyp71a13* double and higher order mutants (dark gray) differ in color from the rest of the genotypes (light gray). Box plots show the median and interquartile range (IQR) of six biological replicates with whiskers at 1.5×IQR. For each replicate, the three youngest fully expanded leaves from one single plant were pooled. Genotypes are indicated below the figure. Statistical analysis was conducted with a one-way ANOVA and a Fisher LSD *post hoc* test with letters indicating significant differences. For camalexin data from mock treated plants, please see **Supplementary Figure 1**.

We next monitored the pathway from IAOx to camalexin in order to benchmark the degree in camalexin deficiency against previous studies with abiotic stimuli. Similar to what was observed for other pathogens (Nafisi et al., 2007; Pastorczyk et al., 2020) and silver nitrate treatment (Müller et al., 2015), lack of *cyp71a13* resulted in a more than 90% reduction in camalexin content at 2 dpi compared to wild type (**Figure 5**, while in response to UV, camalexin content had been reported to be diminished by almost 99% in *cyp71a13* single mutants (Müller et al., 2015). Consistent with all previous studies, loss of both *cyp71a12* and *cyp71a13* resulted in a much stronger reduction in camalexin content to about 0.5% of wild type level (**Figure 5A**). Interestingly, loss of *cyp71b6* in the *cyp71a12/cyp71a13* double mutant background allowed for 4–5% of wild type camalexin content (**Figure 5A**), suggesting that a block in enzymatic conversion of IAN provides a source of building blocks for camalexin biosynthesis. Consequently, the levels of the intermediate downstream of IAN, glutathione-indole-3-acetonitrile (GS-IAN), were comparable between *cyp71a13* single mutants and *cyp71a12/cyp71a13/cyp71b6* triple mutants (**Figure 5B**), with all three mutants showing a 90% reduction in camalexin content (**Figure 5A**). A contribution of the fungus to this phenomenon seems highly unlikely, since other *cyp71a12/cyp71a13* double mutants that were not impaired in *CYP71B6* showed a strong reduction in camalexin content to almost zero (**Figure 5A**).

### *CYP71A13* can compensate for the loss of *CYP71A12* in the biosynthesis of indole carboxylic acid derivatives, but not in indole carboxyaldehyde glucosides

**Figure 6:**
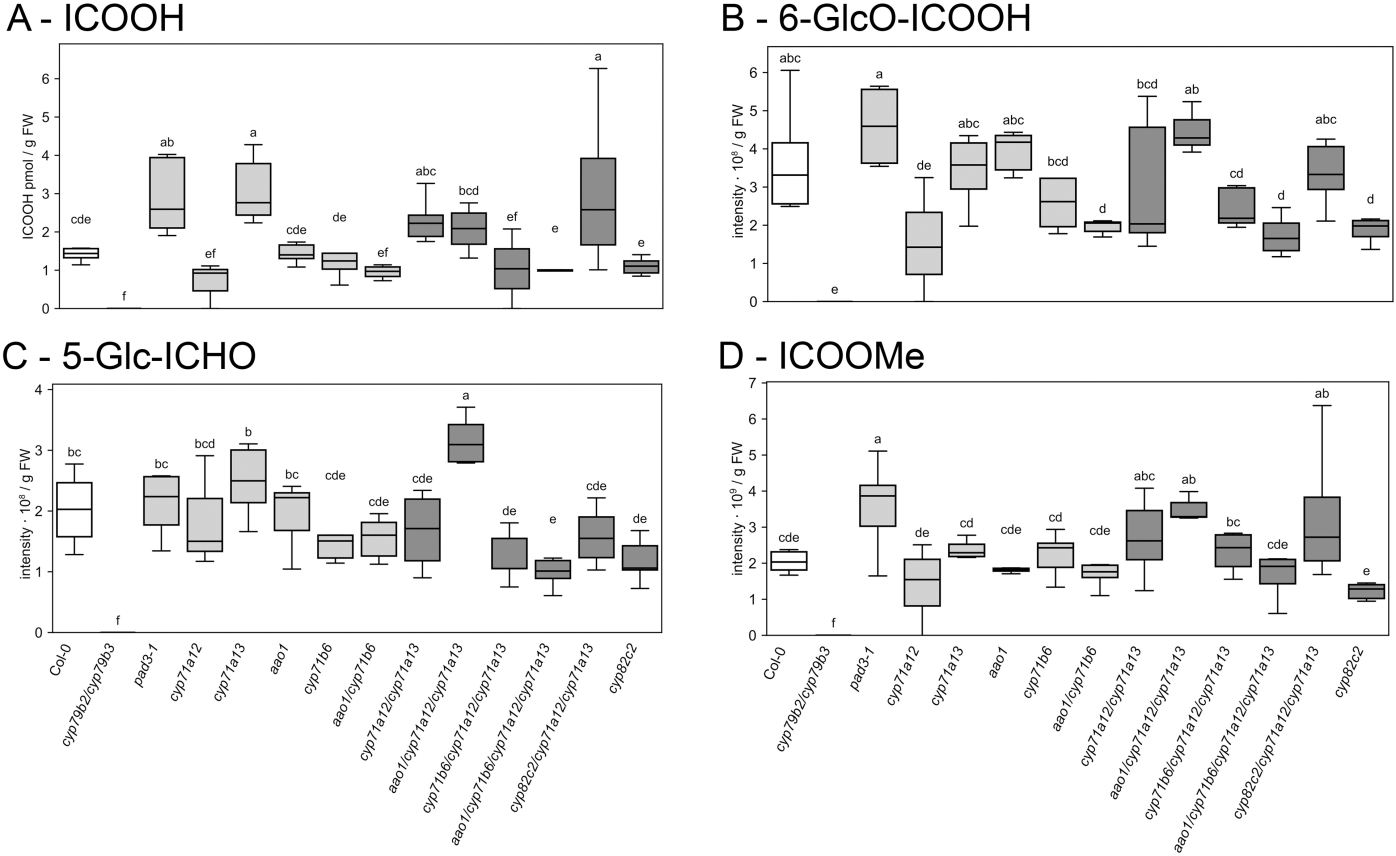
Foliar content of major intermediates of the indole carboxylate branch at 2 days post infection (dpi) with *C. higginsianum*. 4-week-old plants were spray inoculated with 2 · 10^6^ conidia/ml at the end of the light phase and foliar content of **(A)** indole-3-carboxylic acid (ICOOH) **(B)** 6-hydroxyindole-3-carboxylic acid 6-O-β-D-glucoside (6-GlcO-ICOOH), **(C)** indole-3-carbaldehyde 5-O-β-D-glucoside (5-Glc-ICHO) and **(D)** indole-3-carboxylic acid methyl ester (ICOOMe) are shown at two days post infection (dpi). Wild type control (white) and *cyp71a12/cyp71a13* double and higher order mutants (dark gray) differ in color from the rest of the genotypes (light gray). Box plots show the median and interquartile range (IQR) of six biological replicates with whiskers at 1.5×IQR. For each replicate, the three youngest fully expanded leaves from one single plant were pooled. Genotypes are indicated below the figure. Statistical analysis was conducted with a one-way ANOVA and a Fisher LSD *post hoc* test with letters indicating significant differences.

We next examined the branch towards indole carboxylic acids (ICOOH) and indole carboxy nitrile (ICN). Remarkably, the contents of ICOOH, ICHO and ICN derivatives could not be reliably detected in mock conditions and were 50–100-fold lower than at 2 dpi (**Supplementary Figure 2**). In infected leaves at 2 dpi, free ICOOH accumulated in *pad3* and *cyp71a13* relative to wild type at 2 dpi (**Figure 6A**), indicating that only a specific restriction in the camalexin branch leads to increased accumulation of free ICOOH, while this is compensated by additional loss of *CYP71A12*. Interestingly, the *cyp71a12/cyp71a13/cyp82c2* triple mutant showed a similar increase in ICOOH as *pad3* and *cyp71a13*, indicating that reduced flux into free ICOOH caused by *CYP71A12* deficiency can be compensated by a block of ICN biosynthesis (**Figure 6A**). In contrast, 6-(β-glucosyloxy)-indole-3-carboxylic acid (6-GlcO-ICOOH) did not accumulate stronger in any mutant than in the wild type (**Figure 6B**). In *cyp71a12* single mutants and in all mutants lacking both *cyp71b6* and *aao1*, 6-GlcO-ICOOH content was even diminished compared to wild type at 2 dpi, but not in *cyp71a12/cyp71a13* double mutants (**Figure 6B**). Upon challenge with silver nitrate or UV, in contrast, all of the aforementioned mutations decreased 6-GlcO-ICOOH content in an additive manner (Müller et al., 2019).

The accumulation of 5-(β-glucosyloxy)-indole-3-carbaldehyde (5-GlcO-ICHO) upon silver nitrate or UV treatment was decreased in *cyp71b6* or *cyp71a12/cyp71a13* mutants compared to wild type and compensated by additional loss of *aao1* (Müller et al., 2019). At 2 days post *C. higginsianum* infection, almost all *cyp71a12/cyp71a13* double, triple and quadruple mutants showed diminished 5-GlcO-ICHO content (**Figure 6C**). Only loss of *aao1* in the *cyp71a12/cyp71a13* background led to increased accumulation of 5-GlcO-ICHO relative to wild type at 2 dpi (**Figure 6C**). This is consistent with placing AAO1 downstream of IG cleavage by myrosinases and subsequent conversion of IAN to ICHO by CYP71B6 (Böttcher et al., 2014; Müller et al., 2019). An increase in indole-3-carboxylic acid methyl ester (ICOOMe), which serves as a proxy for ICN content (Müller et al., 2019), was also observed in the *cyp71a12/cyp71a13/aao1* triple mutant at 2 dpi (**Figure 6D**). Similarly, this can also be explained by diminished flux of indolic building blocks from IG cleavage into indole carboxylates. Furthermore, the *cyp71a12* single mutant showed reduced ICOOMe levels (**Figure 6D**), corroborating its basal location in the branch towards IAN and ICN, as suggested by previous studies on abiotic stress responses (Müller et al., 2019).

### Altered accumulation patterns of indole carboxylates have no effect on the compatibility towards *C. higginsianum*

**Figure 7:**
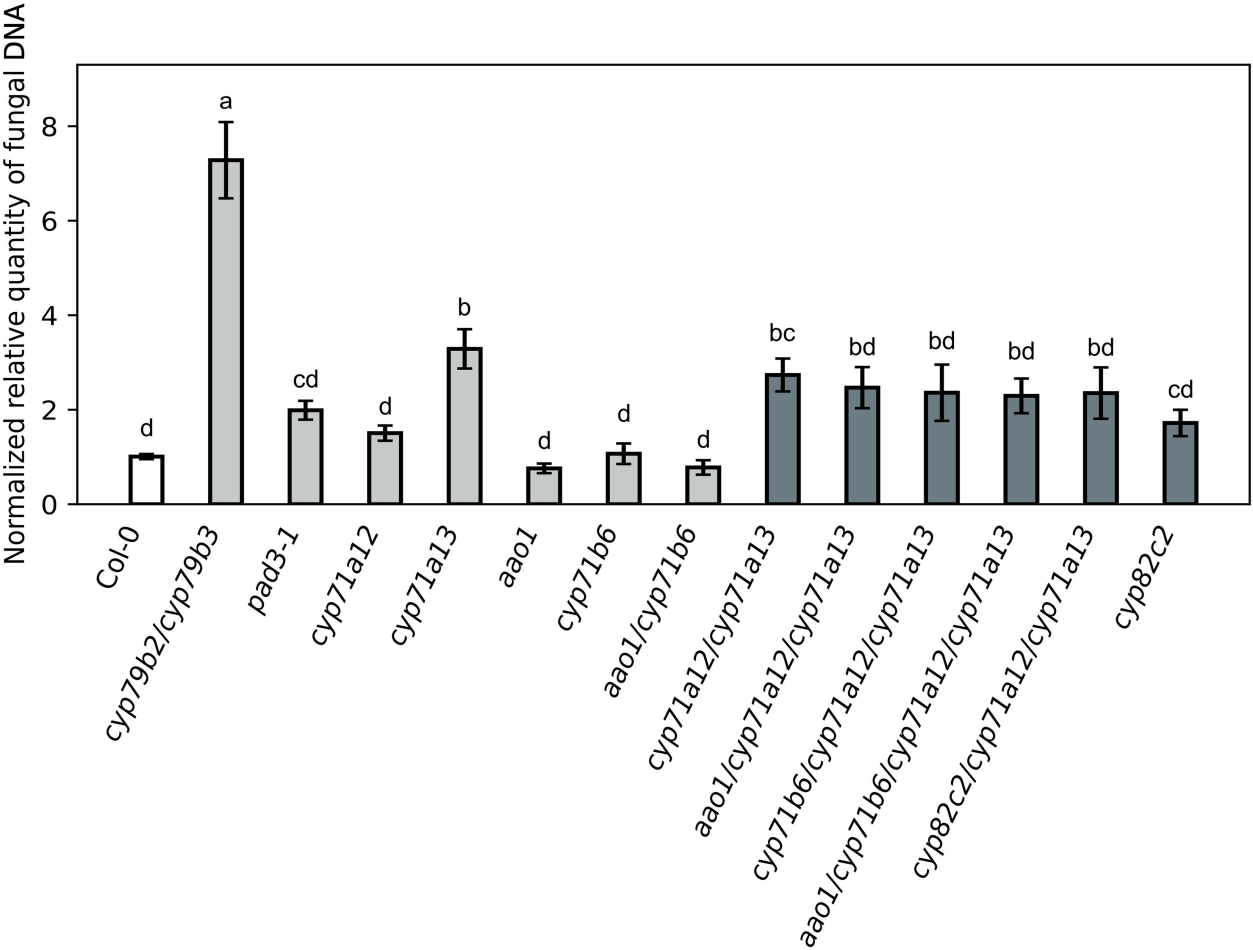
Fungal colonization at 4 days post infection (dpi) after spray infection with *C. higginsianum* as determined by quantitative PCR. 4-week-old plants were spray inoculated with 2 · 10^6^ conidia/ml at the end of the light phase and fungal colonization was determined during the necrotrophic phase at 4 dpi by qPCR. Data are given as normalized relative quantity (NRQ *C.h.* gDNA) relative to Col-0 wild type. Wild type control (white) and *cyp71a12/cyp71a13* double and higher order mutants (dark gray) differ in color from the rest of the genotypes (light gray). Data are means from biological replicates taken from two replicate experiments for *aao1* (N = 10), *cyp71b6* (N = 12), *aao1/cyp71b6* (N = 11), *aao1/cyp71a12/cyp71a13* (N = 12), *cyp71b6/cyp71a12/cyp71a13* (N = 11), *aao1/cyp71b6/cyp71a12/cyp71a13* (N = 13) and *aao1/cyp71a12/cyp71a13* (N = 12), while for the other genotypes biological replicates from up to nine replicate experiments were analyzed: Col-0 (N = 54, 9 Exp), *cyp79b2/cyp79b3* (N = 49, 9 Exp), *cyp71a12* (N = 44, 9 Exp), *cyp71a13* (N = 30, 5 Exp), *cyp71a12/cyp71a13* (N = 41, 8 Exp), *cyp71a12/cyp71a13/cyp82c2* (N = 15, 3 Exp), *cyp82c2* (N = 39, 8 Exp), *pad3* (N = 49, 9 Exp). Error bars indicate the SEM. Genotypes are indicated below the figure. Statistical analysis was conducted with a one-way ANOVA and a Fisher LSD *post hoc* test with letters indicating significant differences.

Given these mutant specific patterns in the accumulation of indolic GSL, camalexin and indole carboxylates, we were curious to examine, how these unique differences translate into effects on compatibility towards the adapted hemibiotrophic fungus *C. higginsianum*. While a complete lack in indolic metabolites in *cyp79b2/cyp79b3* led to an about 8-fold increase in fungal proliferation during the necrotrophic phase at 4 dpi, substantially reduced camalexin accumulation in the *cyp71a13* single and *cyp71a12/cyp71a13* double mutant also resulted in an approx. 3-fold increase in susceptibility towards *C. higginsianum* relative to wild type (**Figure 7**). All higher order mutants in the *cyp71a12/cyp71a13* background did not differ in their susceptibility relative to *cyp71a12/cyp71a13* (**Figure 7**). Fungal colonization in these higher order mutants ranged between 2.2 and 2.4, which compares to 2.7 in the *cyp71a12/cyp71a13* double mutant (**Figure 7**). The camalexin deficient *pad3* mutant also belonged to this group of mutants.

All other mutant genotypes showed significantly less susceptibility towards *C. higginsianum* than *cyp71a12/cyp71a13* and were comparable to wild type (**Figure 7**).

## Discussion

### Only camalexin makes a detectable contribution to the resistance towards *C. higginsianum*

To date, numerous studies have pointed out the importance of indolic glucosinolates and indolic phytoalexins for the defense of Arabidopsis against fungal and bacterial pathogens (see introduction). Loss of the entire indolic secondary metabolism in the *cyp79b2/cyp79b3* mutant resulted in a strong increase in susceptibility towards necrotrophic fungal pathogens like *Alternaria brassicicola* (e.g. Nafisi et al., 2007), *Botrytis cinerea* (e.g. He et al., 2019), or *Plectosphaerella cucumerina* (Frerigmann et al., 2016; Pastorczyk et al., 2020) and hemibiotrophic oomycetes *Phytophthora brassicae* (Schlaeppi et al., 2010), as well as in the loss of non-host resistance towards non-adapted filamentous pathogens (e.g. Hiruma et al., 2010; Pastorczyk et al., 2020). Accordingly, we found that loss of IAOx formation in *cyp79b2/cyp79b3* benchmarked the strongest susceptibility phenotype among the tested mutants in the Arabidopsis–C*. higginsianum* pathosystem (**Figure 7**). However, our study was further set out to refine the individual contributions of the branches downstream of IAOx leading to camalexin, indole carboxylic acid derivatves and indolic glucosinolates to defense against *C. higginsianum*.

Loss of *CYP71A13*, the first step from IAOx towards camalexin biosynthesis, was sufficient to confer increased susceptibility towards *C. higginsianum*. Additional losses of *CYP71A12*, *CYP82C2*, *CYP71B6* and/or *AAO1* in the *cyp71a13* background did not result in significant changes in susceptibility. At the same time, all triple and quadruple mutants deficient in *CYP71A13* and *CYP71A12* did not differ from wild type in their susceptibility, making it impossible to conclude from the genetic point of view, if additional loss of *CYP71B6* and/or *AAO1* have an effect on compatibility. From the biochemical perspective, only quantitative differences in the contents of indole carboxylic acid derivatives between 2- and 3-fold were observed in the analyzed mutants, but no loss of individual compounds (**Figure 6**). Given the quantitative nature of these differences, strong effects on the interaction might not have been expected, even if one of these compounds was biologically active.

The *pad3* mutant, which is deficient in the ultimate steps of camalexin biosynthesis (Zhou et al., 1999; Schuhegger et al., 2006; Böttcher et al., 2009) and accumulates the precursor dihydrocamalexin (DHCA) (Müller et al., 2015) also did not show a significant difference in susceptibility to both, wild type and other camalexin deficient genotypes like *cyp71a13* or *cyp71a12/cyp71a13* that to not accumulate DHCA (**Figure 7**). This leaves the question unresolved, whether DHCA has antimicrobial activity against *C. higginsianum*, as was shown for non-adapted *Plectosphaerella cucumerina* strains (Pastorczyk et al., 2020) and *Pseudomonas syringae* (Kempthorne et al., 2021).

**Figure 8:**
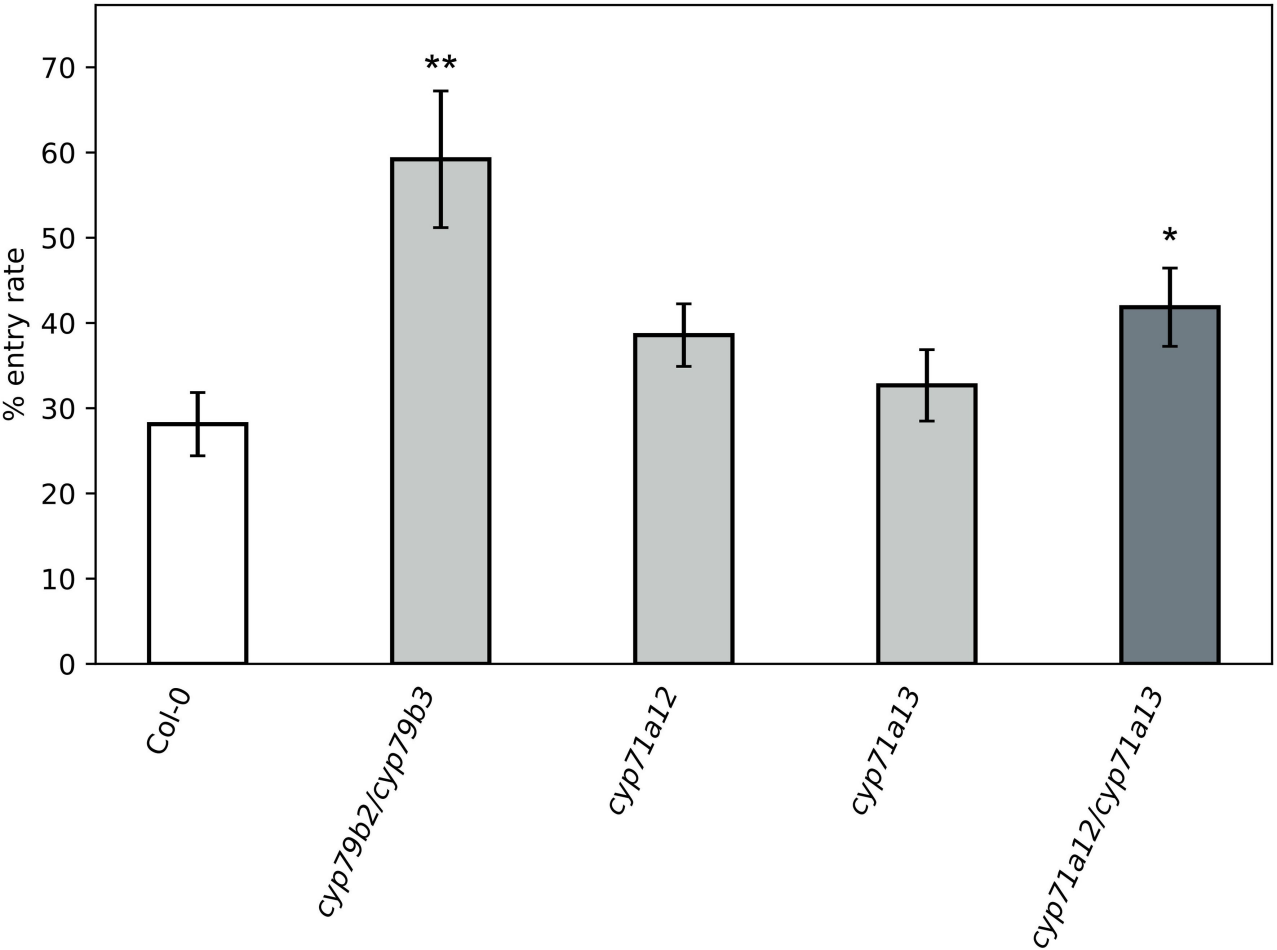
Fungal entry rate scored at 2 days post infection (dpi) after spray infection with *C. higginsianum* by trypan blue staining. 4-week-old plants were spray inoculated with 2 · 10^6^ conidia/ml at the end of the light phase and fungal entry rate was determined during the biotrophic phase at 2 dpi by scoring the developmental stage of approx. 150 infection sites per replicate after histological staining with trypan blue. Values are means of N = 4 for *cyp71a13* and N = 9 biological replicates for all other genotypes ± SEM. Asterisks indicate a significant difference to Col-0 in a Student’s t-test with *p < 0.05 and **p < 0.01.

In comparison to the wild type, the contents of indolic glucosinolates were increased up to 3–4-fold for I3M and around 2-fold for 4MOI3M in all genotypes deficient in *cyp71a13* (**Figure 4**). However, the *cyp71a13* single and the *cyp71a12/cyp71a13* double mutant showed stronger colonization by *C. higginsianum* in the final necrotrophic phase, which indicates that elevated contents of indolic glucosinolates cannot completely compensate for the loss of camalexin in the defense against *C. higginsianum* necrotrophy. To assess if elevated levels of indolic glucosinolates in *CYP71A13* deficient genotypes might support previously described early defense responses that are dependent on PEN2-mediated hydrolysis of IGSL (Bednarek et al., 2009; Clay et al., 2009; Schlaeppi et al., 2010; Frerigmann et al., 2016) and hence, might hamper fungal entry rate, we assessed fungal development in the biotrophic phase at 2 dpi in *cyp71a12* and *cyp71a13* single and double mutants. The *cyp71a12/cyp71a13* double mutant showed an almost twofold increase in *C. higginsianum* entry rate compared to the single mutants and the wild type control (**Figure 8**). While both *cyp71a13* and *cyp71a12/cyp71a13* exhibit a comparable increase in IGSL levels, camalexin deficiency is much more severe in the double mutant, suggesting that the availability of camalexin is more important for hampering early post penetration establishment of *C. higginsianum* than the amount of indolic glucosinolates. We had also previously observed that a 2-fold increase in IGSL content did not affect fungal entry rate in camalexin replete genotypes (Engelsdorf et al., 2017).

Given the fact that camalexin is a classical phytoalexin, which needs to be synthesized *de novo* in response to infection (Glawischnig, 2007), the question arises, if camalexin accumulates in a timely manner to exert an influence on the early biotrophic colonization phase of *C. higginsianum*. At 2 days after infection with *C. higginsianum*, camalexin levels of around 20 ng/cm^2^ are being detectable (Engelsdorf et al., 2013) This is 75-fold less than during necrotrophic colonization at 4 dpi, but fungal colonization increases approx. 400-fold in the same timeframe (Engelsdorf et al., 2013). Provided that camalexin secretion occurs very localized at fungal entry sites (Fuchs et al., 2016), it seems reasonable to assume that local camalexin accumulation is fast enough to affect early fungal pre- and post-penetration development. In addition, the timing of early camalexin accumulation until 48 hpi has been associated with increased resistance towards *C. higginsianum* before (Schmidt et al., 2020). Similarly, camalexin accumulation started as early as 12 hpi after challenge with *P. brassicae* zoospores (Schlaeppi et al., 2010). When placed under the control of chemical induction systems, camalexin accumulation started as early as 6 hpi post induction (Rauhut et al., 2009). However, camalexin accumulation at earlier time points than 2 dpi has never been assessed in this or other studies with *C. higginsianum* (Schmidt et al., 2020), which would be required to demonstrate a direct involvement of camalexin during early post-penetration defense.

Finally, we could not detect that loss of *CYP82C2*, which was shown to be necessary for the production of the phytoalexin 4-OH-ICN (Rajniak et al., 2015), had an effect on the outcome of the interaction, neither in the wild type, nor in the camalexin deficient *cyp71a12/cyp71a13* double mutant background (**Figure 7**). Pastorczyk et al. (2020) had observed that *CYP82C2* contributed to resistance against adapted *P. cucumerina* strains only when both, *PEN2* and camalexin were lacking. In our study, we were unable to assess if comparable roles for *CYP82C2* exist, since we have not included any *pen2* mutant genotypes.

### Comparing the network response to abiotic stress situations

The second aim of our study was to assess the response of indolic secondary metabolism network towards fungal infection in comparison to abiotic stresses like heavy metals and UV light (Böttcher et al., 2014; Müller et al., 2015; 2019). Besides some interesting general observations, we have noticed some minor, but remarkable differences between the responses of the network to abiotic and biotic stress.

As inferred by steady state metabolite levels at 2 dpi, flux into indolic metabolism seems to be higher in response to *C. higginsianum* infection than after challenge with adapted *Pseudomonas* (Stahl et al., 2016) or abiotic stressors. As a first indicator, Trp accumulation in *cyp79b2/cyp79b3* was 30-fold increased at 2 dpi relative to all other genotypes and relative to mock conditions, which compares to a 2–5-fold accumulation of Trp in *cyp79b2/cyp79b3* at 2 dpi after Psm challenge (Stahl et al., 2016) or after silver nitrate or UV treatment (Müller et al., 2015).

Along the same lines, a 3–4-fold increase in I3M was observed in all genotypes lacking *CYP71A13* compared wild type at 2 days after *C. higginsianum* infection (**Figure 4A**). While UV treatment of seedlings resulted in roughly similar numbers, silver nitrate treatment only resulted in about 2-fold accumulation of I3M (Müller et al., 2019). Although the accumulation of 4MOI3M was comparable in all those three treatments, our observations indicate that recognition of the fungal pathogen results in a stronger induction of IGSL biosynthesis than abiotic stimuli. In support of this view, strong induction of IGSL pathway genes has been observed in response to non-adapted *Colletotrichum* species (Hiruma et al., 2010), adapted fungal pathogens (Frerigmann et al., 2016) as well as after PAMP (flg22) treatment (Frerigmann et al., 2016). It is noteworthy that loss of camalexin by itself does not determine increased accumulation of IGSL, as I3M content remained unaltered in the *pad3* mutant (**Figure 4A**). Therefore, partitioning of IAOx between CYP71A12/CYP71A13 and CYP83B1, the committed steps of indolic phytoalexin and IGSL biosynthesis, respectively, seems to govern the accumulation of the respective end products of the individual branches.

Another difference between both examined abiotic stressors, silver nitrate and UV, and *C. higginsianum* infection was that the reduction in camalexin contents in *cyp71a13* single mutants was reduced by 90% after fungal infection, while abiotic stress lead to a 99% reduction in camalexin content in *cyp71a13* (Müller et al., 2019). This indicates that in *cyp71a13*, *CYP71A12* becomes induced much stronger upon challenge with *C. higginsianum* than in response to abiotic stress. In the interaction with *P. cucumerina*, *CYP71A12* was induced 3-fold in the *cyp71a13* mutant, which also showed a 90% reduction in camalexin accumulation (Pastorczyk et al., 2020), similar to what we observed for *C. higginsianum* challenge.

Interestingly, loss of *cyp71b6* in the *cyp71a12/cyp71a13* double mutant background allowed for 4–5% of wild type camalexin content (**Figure 5A**), although loss of the *CYP71* sister gene pair alone should not allow for more than background camalexin formation, which ranges between 1–2% of wild type level (Müller et al., 2015). This observation is consistent with the idea that IAN produced by myrosinase cleavage of IGSL might provide a source of building blocks for camalexin biosynthesis at low rate and we further hypothesize that conversion of IAN to GS-IAN occurs in the presence of GSH involving alternative P450 enzymes. A contribution of *C. higginsianum* to IAN cleavage seems highly unlikely, since other *cyp71a12/cyp71a13* double mutants that were not impaired in *CYP71B6* showed a strong reduction in camalexin content to almost zero (**Figure 5A**).

Free indole carboxylic acid (ICOOH) and its glucosides are present at very low levels in unchallenged plants und accumulate upon abiotic or biotic stress (Böttcher et al., 2014; Frerigmann et al., 2016; Stahl et al., 2016; Müller et al., 2019; Pastorczyk et al., 2020), which would almost fit the definition of a phytoalexin. Nevertheless, ICOOH was described as being a camalexin detoxification product in *Botrytis cinerea* (Kliebenstein et al., 2005; Pedras et al., 2011), suggesting that the accumulation of ICOOH and its glucosides might stem from camalexin as well as IGSL degradation. However, the contents of IGSL and ICOOH were inversely correlated during abiotic challenge (Müller et al., 2019). In fact, the accumulation of ICOOH and 6-GlcO-ICOOH differed strongly between abiotic stress and *C. higginsianum* infection. Upon challenge with silver nitrate or UV, *aao1, cyp71b6*, and the partially redundant *cyp71a12* and *cyp71a13* had additive roles for 6-GlcO-ICOOH accumulation (Müller et al., 2019), while after *C. higginsianum* challenge, only concomitant loss of *AAO1* and *CYP71B6* resulted in reduced 6-GlcO-ICOOH levels (**Figure 6B**). In contrast to the insight gained from abiotic challenges, this indicates that 6-GlcO-ICOOH production in *C. higginsianum* infected leaves is clearly connected to *AAO1* and *CYP71B6* function downstream of IAN and IGSL degradation.

Concerning the source of ICHO derivatives, both previous work on silver nitrate and UV (Müller et al., 2019) are consistent with the current study on fungal challenge, in that loss of *CYP71B6* leads to reduced 5GlcO-ICHO, which indicates that *CYP71B6* converts IAN originating from IG degradation to ICHO derivatives.

## Data Availability

The original contributions presented in the study are included in the article/**Supplementary Material**. Further inquiries can be directed to the corresponding author.

## Supplementary material

Supplementary Table 1 LC-MS/MS parameters used for the idenfitication of indolic metabolites.

Supplementary Figure 1 Foliar content of major indolic and aliphatic glucosinolates in mock conditions.

Supplementary Figure 2 Foliar content of selected indole carboxylate and indole carbaldehyde derivatives in mock conditions compared to 2 dpi.
