# Supplementary material for: Response of the Arabidopsis indolic secondary metabolite network to infection with Colletotrichum higginsianum
Source: Front Fungal Biol. 2026 Mar 16;7:1761006. doi: 10.3389/ffunb.2026.1761006 (PMC13034177; doi:10.3389/ffunb.2026.1761006)
Supplement: Supplementary Table 1 — LC-MS/MS parameters used for the idenfitication of indolic metabolites. [file DataSheet1.pdf]

Supplemental Material

Supplemental Table S1. LC-MS/MS parameters used for the idenfitication of indolic metabolites. RT: retention time. m/z: mass over charge ratio.

**Name**	**elemental composition**	**RT (s)**	**Quant. Ion m/z**	**Method of identification**
5-Glc-ICHO	$C_{15}H_{17}NO_7$	329	324.110	2
6-GlcO-ICOOH	$C_{15}H_{17}NO_8$	405	340.102	2
Camalexin	$C_{13}H_{13}N_3O_2S$	1418	201.048	1
GS-IAN	$C_{20}H_{23}N_5O_6S$	346	462.144	2
ICOOH	$C_9H_7NO_2$	398	162.055	1
ICOOMe	$C_{10}H_9NO_2$	1110	176.076	2
Tryptophan	$C_{11}H_{12}N_2O_2$	382	205.097	1

Method of identification:
1, compound identified by commercial standard;
2, compound annotated by interpretation of mass spectrometry data according to Böttcher et al. (2014).

Supplemental Figures

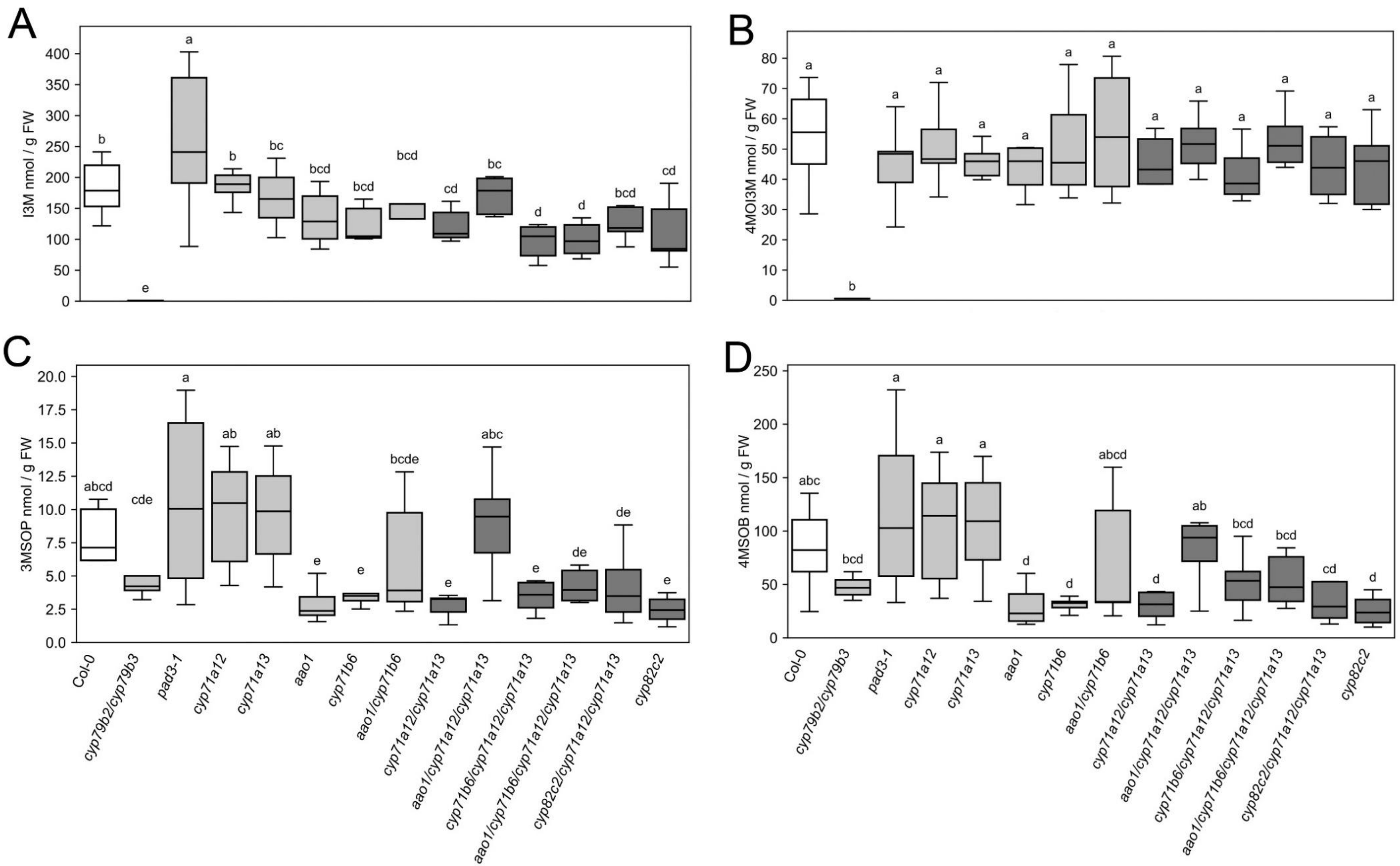

Supplemental Figure S1. Foliar content of major indolic and aliphatic glucosinolates in mock conditions.

Foliar content of the indolic glucosinolates **(A)** I3M and **(B)** 4MOI3M, as well as the two major aliphatic GSL **(C)** 3MSOP and **(D)** 4MSOB from mock treated leaves of four week-old plants are shown at two days post treatment. Wild type control (white) and *cyp71a12/cyp71a13* double and higher order mutants (dark gray) differ in color from the rest of the genotypes (light gray). Box plots show the median and interquartile range (IQR) of six biological replicates with whiskers at 1.5×IQR. For each replicate, the three youngest fully expanded leaves from one single plant were pooled. Genotypes are indicated below the figure. Statistical analysis was conducted with a two-way ANOVA and a Fisher LSD post hoc test with letters indicating significantly different GSL contents.

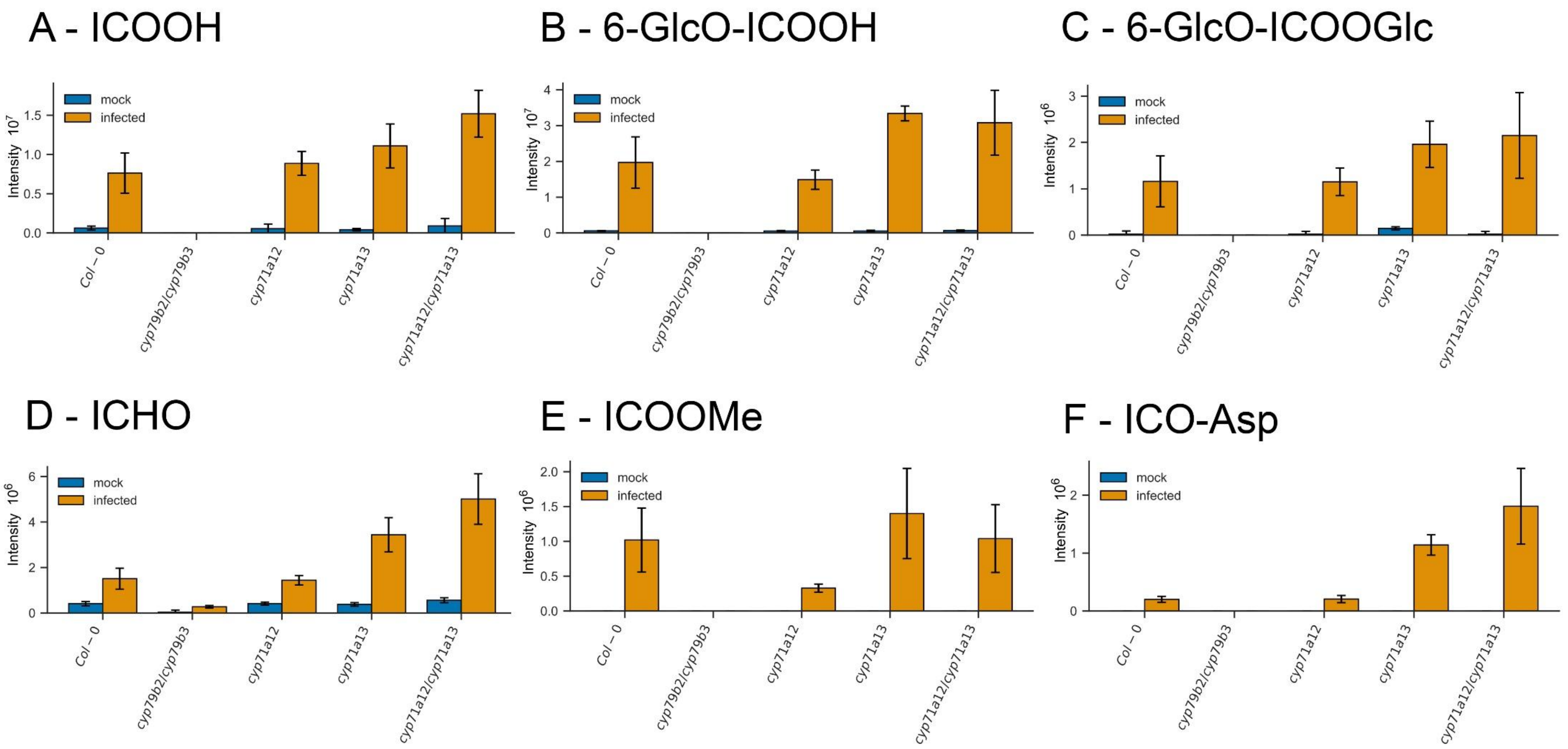

Supplemental Figure S2. Foliar content of selected indole carboxylate and indole carbaldehyde derivatives in mock conditions compared to 2 dpi.

Four week-old plants were spray inoculated with $2 \cdot 10^6$ conidia/ml (orange bars) or water (blue bars) at the end of the light phase and foliar content of **(A)** indole-3-carboxylic acid (ICOOH) **(B)** 6-hydroxyindole-3-carboxylic acid 6-O-β-D-glucoside (6-GlcO-ICOOH), **(C)** β-D-glucosyl 6-hydroxyindole-3-carboxylic acid 6-O-β-D-glucoside (6-GlcO-ICOOGlc), **(D)** indole-3-carbaldehyde (ICHO), **(E)** indole-3-

carboxylic acid methyl ester (ICOOMe) and **(D)** indole-3-carbaldehyde aspartate conjugate (ICO-Asp) are shown at two days post infection (dpi) for the wild type and the indicated mutants. Values are means of four biological replicates ± SEM. For each replicate, the three youngest fully expanded leaves from one single plant were pooled. Genotypes are indicated below the figure.

Supplemental methods

Detection and relative quantification of ICOOH-derivatives shown in the supplemental material was performed essentially as described (Müller et al., 2019), with the following modifications: LC-MS analyses were performed on a bio-inert 1290 series UHPLC system which was interfaced to a Q-TOF mass spectrometer (G6546AA, Agilent Technologies) via a dual Agilent jet stream electrospray ion source. MassHunter LC/MS Data Acquisition and Qualitative Analysis software was used for data acquisition and data evaluation, respectively. The mass spectrometer was operated in low mass range (*m/z* 1700), Gas temp 200°C, VCap 3000 V. The instrument was auto tuned and calibrated according to manufacturer´s recommendations using ESI-L tuning mix (Agilent Technologies). Extracts (2 µL and 5 µl injection volume for positive and negative ionisation, respectively) were separated on a Zorbax RRHD Eclipse Plus C18 column (100 × 2.1 mm, 1.8 µm particle size, Agilent Technologies) using 0.1% (v/v) formic acid in water and 0.1% (v/v) formic acid in MeOH as eluent A and B, respectively. The following binary gradient program at a flow rate of 500 µL min^{-1} was applied: 0–15 min, linear from 5 to 80% B; 15–15.1 min, linear from 80 to 95% B; 15.1–18 min, isocratic, 95% B; 18–20.5 min, isocratic, 5% B. The column temperature was maintained at 40°C and the autosampler temperature at 8°C. Eluting compounds were detected from m/z 70–1000. Mass spectra were acquired in centroid mode using an acquisition rate of 2.5 spectra per second.
